# A simple novel device for air sampling by electrokinetic capture

**DOI:** 10.1186/s40168-015-0141-2

**Published:** 2015-12-27

**Authors:** Julian Gordon, Prasanthi Gandhi, Gajendra Shekhawat, Angel Frazier, Jarrad Hampton-Marcell, Jack A. Gilbert

**Affiliations:** Inspirotec LLC, 3307 Meadow Lane, Glenview, IL 60025 USA; Department of Materials Science and Engineering, McCormick School of Engineering and Applied Science, Northwestern University, 2220 Campus Drive, #2036, Evanston, IL 60208 USA; Genomic and Systems Biology, Bioscience Division, Argonne National Laboratory, 9700 South Cass Avenue, Argonne, IL 60439 USA; Department of Ecology and Evolution, University of Chicago, 1101 E 57th Street, Chicago, IL 60637 USA; Department of Surgery, University of Chicago, 5841 South Maryland Avenue, MC 5029, Chicago, IL 60637 USA; Marine Biological Laboratory, 7 MBL Street, Woods Hole, MA 02543 USA; College of Environmental and Resource Sciences, Zhejiang University, Hangzhou, 310058 China

**Keywords:** Atomic force microscopy, Reverse transcriptase PCR, Air sampling, Field study, Aerosol, Nanoparticles, Aerobiome, Amplicon sequencing, Bacteria, Molds

## Abstract

**Background:**

A variety of different sampling devices are currently available to acquire air samples for the study of the microbiome of the air. All have a degree of technical complexity that limits deployment. Here, we evaluate the use of a novel device, which has no technical complexity and is easily deployable.

**Results:**

An air-cleaning device powered by electrokinetic propulsion has been adapted to provide a universal method for collecting samples of the aerobiome. Plasma-induced charge in aerosol particles causes propulsion to and capture on a counter-electrode. The flow of ions creates net bulk airflow, with no moving parts. A device and electrode assembly have been re-designed from air-cleaning technology to provide an average air flow of 120 lpm. This compares favorably with current air sampling devices based on physical air pumping. Capture efficiency was determined by comparison with a 0.4 μm polycarbonate reference filter, using fluorescent latex particles in a controlled environment chamber. Performance was compared with the same reference filter method in field studies in three different environments. For 23 common fungal species by quantitative polymerase chain reaction (qPCR), there was 100 % sensitivity and apparent specificity of 87 %, with the reference filter taken as “gold standard.” Further, bacterial analysis of 16S RNA by amplicon sequencing showed equivalent community structure captured by the electrokinetic device and the reference filter. Unlike other current air sampling methods, capture of particles is determined by charge and so is not controlled by particle mass. We analyzed particle sizes captured from air, without regard to specific analyte by atomic force microscopy: particles at least as low as 100 nM could be captured from ambient air.

**Conclusions:**

This work introduces a very simple plug-and-play device that can sample air at a high-volume flow rate with no moving parts and collect particles down to the sub-micron range. The performance of the device is substantially equivalent to capture by pumping through a filter for microbiome analysis by quantitative PCR and amplicon sequencing.

## Background

Understanding of the microbiology of air, the aerobiome, is an emerging field of discovery. High-throughput sequencing methods are being used to explore the spatiotemporal distribution of bacterial and fungal populations [1–10]. A variety of sampling methods have been used for studying the air microbiome [3, 11–14]. A variety of different sampling devices are currently available to acquire air samples of microbial and viral particles [15]. These technologies include filters, impingers, impactors, and wet or dry cyclones. The underlying principle of impactors, impingers, and cyclones is the use of an abrupt change in direction of airflow so that aerosol particles will continue on to a surface by virtue of their momentum. Filters are microporous membranes, impingers capture on to the surface of a nutrient agar plate for subsequent colony counts, and impactors capture on a solid surface for subsequent elution, as do dry cyclones. Wet cyclones capture by vortexing into a liquid phase. Aerosol particles may also be separated into size classes with multi-stage devices. For existing devices, capture efficiency falls off rapidly with particle size and there is considerable variability in performance [16–18]. All of these devices require pumping against some resistance. Different apparent microbial communities were found from the use of different air sampling techniques [11, 13]. There is thus a need in aerobiome analysis for a sampling procedure that does not bias the measured biodiversity. Here, we introduce the use of a very simple device for collection of samples and show equivalence to a reference method using filtration. In addition, a variety of air sampling methods have been applied to the airborne transmission of disease [19–30].

Brown first described the principle of ionic propulsion in US patents [31, 32]. A corona wire is subject to a high voltage, creating plasma that imparts charge on particles in the vicinity. The charged particles are then propelled by the voltage gradient to electrodes at an opposing potential. The net flow of charged particles imparts forward momentum on the surrounding medium. The result is a net airflow with no moving parts. This principle has been used in commercial air-cleaning devices [33]. Custis et al. [34] used such an air-cleaning device for collecting dust from the air for measuring allergens. We have developed a mini-scale device using the same principle with optimized airflow and an electrode cartridge that is optimized for sample collection (Inspirotec Sampler). We have demonstrated its use for detection of allergens by immunoassay [35, 36] and viruses by quantitative PCR [37]. The device is simple to operate, compact, and can be placed unobtrusively in any environment. Here, we compare performance with a filter reference method for analysis of the aerobiome.

## Results

For the purpose of this study, three environments were selected for side-by-side comparison of the Inspirotec Sampler, with an air filter as a reference device. The environments were a clean bathroom, a basement room with an exposed sump drain, and a hay storage room in a large equestrian facility.

### Mold spores

**Fig. 1:**
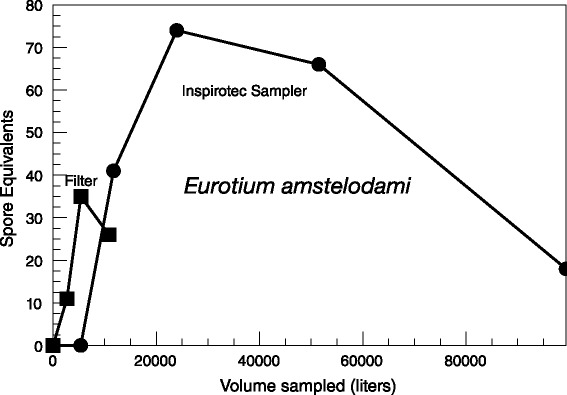
Time course for spore collection. Air samples were taken at 0, 45, 90, 180, 360, and 720 min for the Inspirotec Sampler and at 0, 180, 360, and 720 min for the filters in the stable of Table 1. Volume sampled is computed from the individual Inspirotec Sampler flow rates and from the 15 lpm setting for the filters. Zero time samples were placed in the respective device, power was not turned on, and they were otherwise treated identically to the timed samples

Mold spores are ubiquitous in the environment but vary according to temperature, humidity, season, and other environmental conditions. Table 1 shows the results of 24-h samples in the three locations. The Inspirotec Sampler values have not been corrected for capture efficiency (approximately 20 %, see “Methods” section). If this correction were applied, the values would all be consistent within the standard deviations achievable by the quantitative polymerase chain reaction (qPCR). Those cases where the species was detected by both methods are indicated by green shading. In no instance was a species detectable by the filter and not detectable by the Inspirotec Sampler. In eight instances, the Inspirotec Sampler detected a species that was not detected by the filter, indicated by yellow shading in Table 1. By this criterion, if the filter is considered a gold standard, the sensitivity is 100 % and the specificity is 87 %. However, these are likely to be true positives since the Inspirotec Sampler is sampling a larger volume of air in a given time. To address this concern, we examined the accumulation rate for *Eurotium amstelodami* on both filters and the Inspirotec Sampler (Fig. 1). This shows that the Inspirotec Sampler processed both a larger volume, and correspondingly, a larger quantity of spore equivalents were captured, when compared to the filter. As with Table 1, these numbers have not been corrected for capture efficiency. It is not clear why the quantity of spore equivalents captured appears to peak at 6 h for both filter sampling and Inspirotec Sampler. Nevertheless, this illustrates the advantage of the high sampling volume of the Inspirotec Sampler. This advantage is compounded by the Inspirotec Sampler’s easier logistical set-up and silent performance.

**Table 1:**
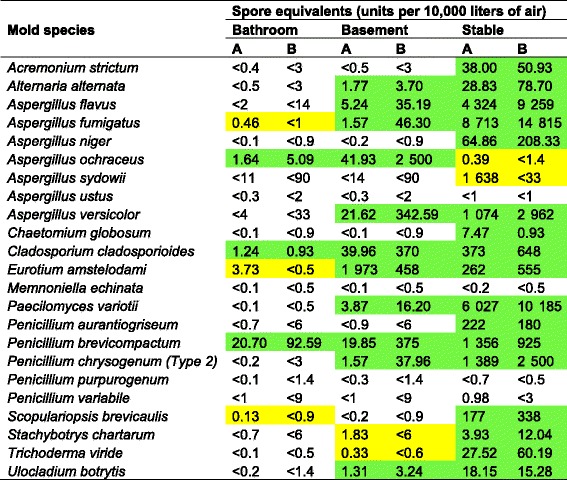
Mold spores in three locations

*A* Inspirotec Sampler, *B* filter. Spore equivalent values were determined by multiplex qPCR (see “Methods” section). Green shading: detectable by both methods; yellow: detectable by Inspirotec Sampler only; no shading: below the limit of detection of the qPCR

### Bacterial diversity

**Fig 2:**
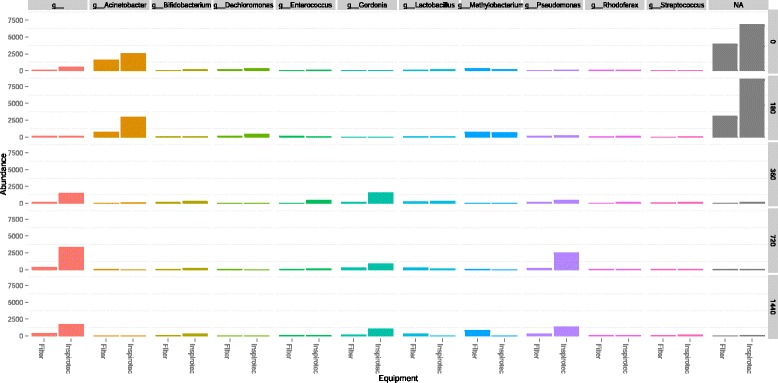
Relative abundance of bacterial genera as a function of sampling time. Samples were collected following the time protocol of Fig. 1 in the basement location of Table 1. The top 25 sequences were selected from the OTU table, and relative abundance of bacterial genera was plotted across consecutive time points between samplers as described in the “Methods” section*.* Zero time samples were as in Fig. 1

Timed samples were run in the basement environment with the same schedule as in Fig. 1. Bacterial 16S rRNA amplicon sequencing generated a total of 1,294,310 sequences from 22 samples. When rarified to 9800 sequences per sample, 385,076 operational taxonomic units (OTUs; 97 % identity) were identified. No significant difference in microbial community structure was observed between the Inspirotec Samplers and the reference method with the use of the R Project for Statistical Computing freeware (weighted or unweighted UniFrac distance ADONIS, *p* > 0.05, *R* = 0.06). False-discovery rate (FDR) and Bonferroni-corrected *p* values showed no significant differences in OTU frequencies between platforms. The genus-level community profile generated by both technologies comprised predominantly *Acinetobacter*, *Gordonia*, *Methylobacterium*, and *Pseudomonas* (Fig. 2). Differences in abundances in Fig. 2 are therefore not significant.

**Fig 3:**
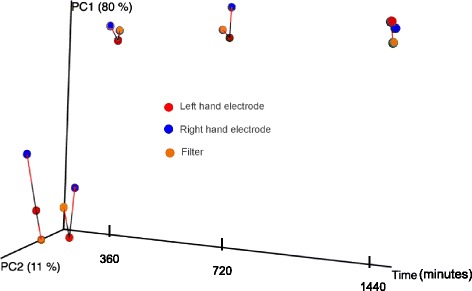
Procrustes analysis of bacterial community structure as a function of sampling time. A principal coordinate analysis (PCoA) was generated for each of the replicate electrodes and the filter samples. CV1 and CV2 are the first two dimensions of variance that describe the most variance in the multidimensional structure of this population, represented by the percentages on the axes. UniFrac distances from each PCoA were comparatively superimposed by collection time

Interestingly, 180 min produced a signal highly similar to the time zero (blank) suggesting that this time frame was insufficient to generate enough biomass for the detection threshold of the amplicon sequencing technology (Fig. 2). However, by 360 min, the community profiles were significantly different from time zero. Reagent-based contamination is known to be an issue [38] and explains the detected signal for blank and 180 min. The significance of the similarity between microbial profiles generated by the filter and Inspirotec Sampler technologies at each time point was assessed using Procrustes analysis including the left and right electrode (technical replicates) of the Inspirotec Sampler as well as the pump-driven filter. Over the course of time, there was no significant difference between either technical replicate or the air filter, despite greater variability between samplers at the starting zero time (Monte Carlo, *p* > 0.05) (Fig. 3).

### Particle size and capture

**Fig 4:**
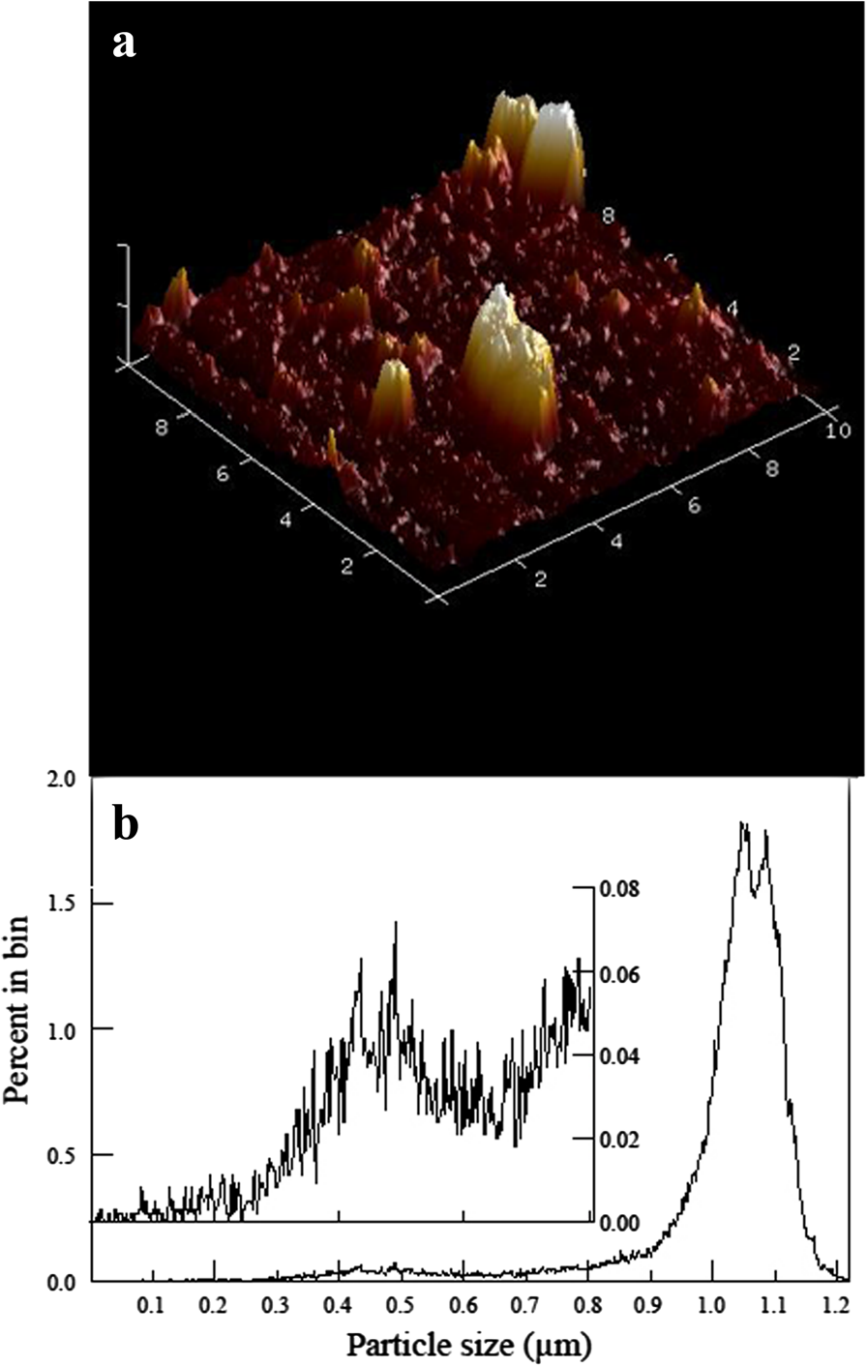
Atomic force microscopy of biomass captured. The Inspirotec Sampler was run 6 days in the bathroom (Table 1) and scanned (see “Methods” section). **a** False color 3D representation of a 10 × 10 μm square. Height above the plane is in the same micrometer scale. **b** Particle size distribution analysis of same data. Size distribution in 512 bins and percent of particles in each bin plotted. *Inset*: 0–0.8 μm range shown on ×10 expanded scale

A prediction of the method of using ionic propulsion to capture the charge particles is that the capture should be independent of particle size of mass, unlike current aerodynamic-based sampling systems. The location of capture is dependent on the force vectors determined by the voltage gradient. Mass may affect particle acceleration and velocity, but the final capture location is determined by a potential well. We therefore explored sample capture with a random sample of air (bathroom of Table 1) and examined the size distribution of captured particles by atomic force microscopy. Particles down to the nanometer range were captured (Fig. 4), as demonstrated using a visual representation of particle size density (Fig. 4a), as well as a size distribution curve, with a significant fraction trailing into the lower range (Fig. 4b).

The Inspirotec Sampler was run in the bathroom for 24-h (Table 1) and scanned (see “Methods” section). The inset shows the lower end of the distribution curve and captured particles extending down into the 500-nm range and below. The sampler is thus able to capture particles going down to very low sizes and in the range that will penetrate the lungs and cause symptoms. This illustrates the range of sizes of not-identified aerosol particles that are captured.

## Conclusion

We have demonstrated the applicability of an electrokinetic air sampler for the molecular detection of microorganisms in air. All procedures are of wide applicability to any measurements in the aerobiome. This technology is easily deployed as it can be plugged into any electrical socket, silent, has low visual impact, and so could be readily applied to indoor settings for identifying and tracking emerging pandemics. The device performance was comparable to or exceeded that of the reference method.

## Discussion

The device is inexpensive and requires no technical skill to operate, compared with any other competing technology. Both SARS and MERS [39, 40] epidemics may be traced back to human-animal interfaces, and early deployment in such emerging pandemics would facilitate the tracing of early stages and subsequent routes of transmission. In a separate study [37], we showed the capability of capturing Venezuelan equine encaphilitis virus in a controlled environment chamber at the US Army Edgewood Chemical Biological Center with aerosol particles down to 1 μm. The viruses had been inactivated by gamma irradiation. The result was that a large proportion of the original virions had RNA that was not amplifiable, so the capture efficiency was apparently very low based on the original virus titer. However, analysis by digital PCR using the Poisson distribution at low amplicon concentrations showed that the capture efficiency was in the range of 20–40 % for these articles. Here, the performance exceeded that of the reference method using a microporous filter and showed ability to detect mold spores using EPA-accepted PCR technology [41]. Of the species for which primers and probes were used in the qPCR, *Acremonium strictum*, *Alternaria alternata*, *Aspergillus flavus*, *Aspergillus fumigatus*, *Aspergillus niger*, *Aspergillus ochraceus*, *Aspergillus sydowii*, *Aspergillus ustus*, *Aspergillus versicolor*, *Chaetomium globosum*, *Cladosporium cladosporioides*, *Eurotium amstelodami*, *Memnoniella echinata*, *Paecilomyces variotii*, *Penicillium aurantiogriseum*, *Penicillium brevicompactum*, *Penicillium chrysogenum* (type 2), *Penicillium purpurogenum*, *Penicillium variabile*, *Scopulariopsis brevicaulis*, *Stachybotrys chartarum*, *Trichoderma viride*, *Ulocladium botrytis*, all but *Aspergillus ustus*, *Memnoniella echinata*, and *Penicillium variabile* were detectable as spores over the three locations tested.

Another feature of the electrokinetic propulsion is the ability to capture and measure particles down into the nanoparticle range. Particles generated from respiratory activities in the range of 0.05 to 500 μm are associated with infection [42]. Most commonly used air sampling devices have a cut-off at about 1 μm [15]. Allergens may extend down to a size range that has been missed by current sampling technology [43], and there is evidence that bacterial endotoxin, which exacerbates the effect of allergens, may exist in size ranges below the size of bacteria [44]. The device described in this publication will have the capability of extending the size range of particles that can be collected from the aerobiome.

Fahlgren et al. [13] found that the diversity of microbial communities captured by different samplers in external environments in Norway and Sweden were similar, whereas Hoisington et al. [11] have found significant inconsistencies between different sampler types at locations within a US retail store. In neither case was consideration made to the time of run needed to resolve reagent background. We showed that a minimum of 6 h sampling was required regardless of the method. We demonstrate the application of ionic propulsion technology to capture a wider range of particle sizes than with traditional air filter sampling, with no significant bias in fungal and bacterial community recovery.

## Methods

Inspirotec Samplers and accompanying electrode cartridges were provided by Inspirotec LLC (Glenview, IL). A commercial air cleaner was modified to achieve a uni-directional flow. The disposable capture cartridges were designed to optimize capture of aerosol particles and for easy release into extraction tubes. They were made by 3D printing at Exact Prototyping, Joliet, IL, and stainless steel electrode strips by Lakeshore Cutting Solutions, Zeeland, MI. Electrodes were finished by Able Electropolishing, Chicago, IL. Cartridges were washed with 70 % isopropanol and air dried, and electrodes were washed with isopropanol and dried in a vacuum oven for 30 min at 180 °C. Assembled cartridges and electrodes were heat-sealed into polyethylene bags until use. Once the electrodes have been released, they cannot be re-used. There is no potentially non-sterile surface that physically contacts the electrodes between removal from the bag and release into the collection tube. An updated version of the Inspirotec Sampler has a block of honeycomb-structured MnO_2_ catalyst covering the outlet. This modification removes traces of ozone from the effluent air but does not affect performance. Samplers and cartridges can be purchased by sending an email to the senior author with heading “research request.” The sampler will have a lower cost than any alternative system.

Flow rates were measured with a hot wire anemometer by averaging measured flow velocities across the width of a duct placed over the outlet [45]. Individual samplers’ flow rates varied by 130 ± 13 lpm. Capture efficiency (determined by AlburtyLabs, Inc., Drexel, MO) as judged by capture of 1 μm Fluoresbrite® YG carboxylate microspheres (Polysciences, Inc., Warrington, PA) in a controlled environment chamber, compared with capture by a reference sampler consisting of a 0.22-μM polycarbonate filter, was (23 ± 5)% based on 16 determinations. Fluorescence of captured microspheres was with a Turner Quantech fluorometer. The reference samplers were run at 14 lpm. Thus, the capture efficiency is more than compensated by the higher volumes of air sampled.

Following their standard isolation procedure, captured mold spores were determined by qPCR for the 23 most common mold species by EMLab P&K, Marlton, NJ. Primers and probes and amplification conditions used are in [41]. Electrodes were released into 15 ml Falcon tubes and shipped overnight for analysis. They were extracted by vortex mixing intermittently over 10 min with 0.05 % Tween 20, and spores were centrifuged down and extracted by bead-beating [46]. Results were computed as spore equivalents [46–49]. These publications show that standard deviations of spore equivalent counts may be ±1 log or more.

Amplicon sequencing: each cartridge holds two electrodes. Left and right electrodes were processed separately, and so were effective duplicates. Individual electrodes were placed into sterile 15-ml conical tubes with 1 ml of sterile water. Samples were extracted for 1 min on a Vortex Genie (MO BIO Laboratories, Inc., Carlsbad, CA). Their standard PowerSoil extraction was performed according to the manufacturer’s suggested protocol with the addition of a 20-min incubation at 65 °C after addition of solution C1, as suggested by the Earth Microbiome Project. Genomic DNA was amplified using the Earth Microbiome Project barcoded primer set, adapted for MiSeq (Illumina Inc., San Diego, CA) by adding nine extra bases in the adapter region of the forward amplification primer that support paired-end sequencing. The V4 region of the 16S rRNA gene (515F-806R) was amplified with region-specific primers that included the Illumina flow cell adapter sequences. The reverse amplification primer also contained a 12-base barcode sequence that supports pooling of up to 2167 different samples in each lane. Each 25-μl PCR reaction contains 12 μl of MO BIO PCR water (certified DNA-free), 10 μl of 5 Prime HotMasterMix (5 μM concentration), 1 μl of forward primer (5 μM concentration, 200 pM final), 1 μl Golay barcode-tagged reverse primer (5 μM concentration, 200 pM final), and 1 μl of template DNA. The conditions for PCR were as follows: 94 °C for 3 min to denature the DNA, with 35 cycles at 94 °C for 45 s, 50 °C for 60s, and 72 °C for 90s; and with a final extension of 10 min at 72 °C to ensure complete amplification. PCR amplifications were completed in triplicate and then pooled. Following pooling, amplicons were quantified using PicoGreen (Invitrogen) and a plate reader. Once quantified, different volumes of each of the products were pooled into a single tube so that each amplicon was represented equally. This pool was then cleaned using the UltraClean® PCR Clean-Up Kit (MO BIO) and quantified using Qubit (Invitrogen). Sequencing of the prepared library was performed on the Illumina MiSeq platform, using the sequencing primers and procedures described in the supplementary methods of Caporaso et al. [50].

For the atomic force microscopy, a sampler was run for 6 days in the clean bathroom location of Table 1. It was examined with atomic force microscopy with a Bruker dimension Icon system which has the capability of providing sub-nanometer resolution. Imaging was done in tapping mode with super sharp silicon probes. Results were analyzed with the Bruker analysis software.

### Availability of supporting data

All sequence data will be made available through FigShare, http://dx.doi.org/10.6084/m9.figshare.1603492
